# Dietary intake of glucoraphanin during pregnancy and lactation prevents the behavioral abnormalities in the offspring after maternal immune activation

**DOI:** 10.1002/npr2.12112

**Published:** 2020-05-28

**Authors:** Yuko Fujita, Atsuhiro Fujita, Tamaki Ishima, Ayumi Hirai, Shigenori Suzuki, Hiroyuki Suganuma, Kenji Hashimoto

**Affiliations:** ^1^ Division of Clinical Neuroscience Chiba University Center for Forensic Mental Health Chiba Japan; ^2^ Innovation Division Kagome Co., Ltd. Nasushiobara Japan

**Keywords:** autism, glucoraphanin, nutrition, prevention, schizophrenia, sulforaphane

## Abstract

**Aim:**

Epidemiological data suggest that maternal immune activation (MIA) plays a role in the etiology of neuropsychiatric disorders including autism spectrum disorder (ASD) and schizophrenia. However, there is no prophylactic nutrition that can prevent the onset of neurodevelopmental disorders in offspring after MIA. The aim of this study was undertaken to examine whether dietary intake of glucoraphanin (GF: the precursor of a natural anti‐inflammatory compound sulforaphane) can prevent the onset of behavioral abnormalities in offspring after MIA.

**Methods:**

One percent of GF food pellet or normal food pellet was given into female mice during pregnancy and lactation (from E5 to P21). Saline (5 mL/kg/d) or poly(I:C) (5 mg/kg/d) was injected into pregnant mice from E12 to E17. Behavioral tests and immunohistochemistry of parvalbumin (PV) were performed in male offspring.

**Results:**

Dietary intake of GF during pregnancy and lactation prevented cognitive deficits and social interaction deficits in the juvenile offspring after MIA. Furthermore, dietary intake of GF during pregnancy and lactation prevented cognitive deficits in the adult offspring after MIA. Moreover, dietary intake of GF prevented the reduction of PV immunoreactivity in the medial prefrontal cortex of adult offspring after MIA.

**Conclusion:**

These data suggest that dietary intake of GF during pregnancy and lactation could prevent behavioral abnormalities in offspring after MIA.

## 1 INTRODUCTION

Epidemiological data suggest that the prenatal environmental factors, including maternal immune activation (MIA), contribute to the onset of neuropsychiatric disorders such as autism spectrum disorder (ASD) and schizophrenia in offspring.^1^, ^2^ There are a number of publications showing associations between maternal inflammatory biomarkers and these disorders.^2^ Meta‐analyses suggest that maternal infection during pregnancy increases the risk of these disorders in offspring.^3^, ^4^ Importantly, there are accumulating interests in the early prevention by anti‐inflammatory compounds.^5^, ^6^, ^7^ However, there are no anti‐inflammatory compounds that can be used in the early intervention for pregnant women with MIA. Polyriboinosinic‐polyribocytidilic acid (poly[I:C]), a Toll‐like receptor 3 agonist, is widely used as an animal model of MIA.^2^, ^8^, ^9^, ^10^, ^11^, ^12^, ^13^, ^14^

The Nuclear factor erythroid 2‐related 2 (Nrf2) is a transcription factor which plays a crucial role in attenuating oxidative stress and inflammation.^15^, ^16^ Sulforaphane (SFN) is a naturally occurring compound with potent anti‐inflammatory effects. In addition, glucoraphanin (GF), glucosinolate precursor of SFN, is found in cruciferous vegetables.^17^ SFN attenuated abnormal behaviors in rodents after the administration of phencyclidine (PCP).^18^ Furthermore, the supplementation of GF during juvenile and adolescent stages prevented the behavioral abnormalities in adult mice after repeated PCP administration^19^ or MIA.^12^ These findings suggest that supplementation of GF may have prophylactic effects for neuropsychiatric disorders such as schizophrenia.^7^ However, there are currently no reports showing that supplementation of GF in pregnant rodents can affect the development of abnormal behaviors in juvenile and adult offspring after MIA.

This study was undertaken to investigate whether dietary intake of GF food pellets during pregnancy and lactation could attenuate the development of abnormal behaviors in juvenile and adult offspring after MIA. Furthermore, we performed parvalbumin (PV)‐immunohistochemistry since the reduction of PV immunoreactivity in the mPFC is associated with neuropsychiatric disorders.^14^, ^20^, ^21^

## 2 MATERIALS AND METHODS

### 2.1 Animals

Pregnant ddY mice (embryo at the 5th day [E5], 9‐10 weeks old) were obtained from Japan SLC Inc. Pregnant mice were caged into individually clear polycarbonate cage (22.5 × 33.8 × 14.0 cm) under a controlled 12/12h light‐dark cycle (lights on from 07:00 am to 07:00 pm), with room temperature at 23 ± 1°C and humidity at 55 ± 5%. All mice had ad libitum access to water and food pellets. The experimental procedure using animals was approved by the Chiba University Laboratory Animal Care and Use Committee (permission number: 28‐272).

### 2.2 Preparation of 0.1% GF and prenatal injection of poly(I:C)

Food pellets (CE‐2; Japan CLEA, Ltd.) containing 0.1% glucoraphanin (GF) were prepared as reported previously.^15^, ^19^, ^22^, ^23^, ^24^ Normal food pellet or 0.1% GF food pellet was given to female mice during pregnancy and lactation (from E5 to P21 [weaning]). Subsequently, normal food pellets were given to all offspring mice from P21 to behavioral tests or PV immunohistochemistry.

The schedule of prenatal poly(I:C) treatment was performed as reported previously.^8^, ^9^, ^10^, ^11^, ^12^ The pregnant mice were injected intraperitoneally (i.p.) for six consecutive days from E12 to E17 with poly(I:C) (5.0 mg/kg/d, Sigma‐Aldrich Co. Ltd.) or an equivalent saline (5 mL/kg). The male offspring were separated from their mothers at P21, and mice were caged each three to five in the groups.

### 2.3 Behavioral analysis

The novel object recognition test (NORT) and the three‐chamber social interaction test were performed as reported previously.^8^, ^9^, ^10^, ^11^, ^12^, ^21^

### 2.4 PV immunohistochemistry

Parvalbumin immunohistochemistry using mouse polyclonal antiparvalbumin (PV) antibody (1:100; abcam, ab11427) was performed as reported previously.^8^, ^9^, ^10^, ^11^, ^12^, ^21^ The staining intensity of PV immunoreactivity in the inflalimbic (IL) and prelimbic (PrL) regions of mPFC was analyzed using a light microscope equipped with a CCD camera (Olymups IX70) and the SCION IMAGE software package. Images of sections (n = 4 for each mouse) within mPFC region were captured using a 100 × objective with a Keyence BZ‐X700 microscope (Keyence Corporation).

### 2.5 Statistical analysis

All data are shown as mean ± standard error of the mean (SEM). The data were analyzed by two‐way analysis of variance (ANOVA), followed post hoc Bonferroni test. Significance for results was set at *P* < .05.

## 3 RESULTS

### 3.1 Effects of dietary intake of 0.1% GF food pellets during pregnancy and lactation on behavioral abnormalities in the juvenile offspring after MIA

**Figure 1:**
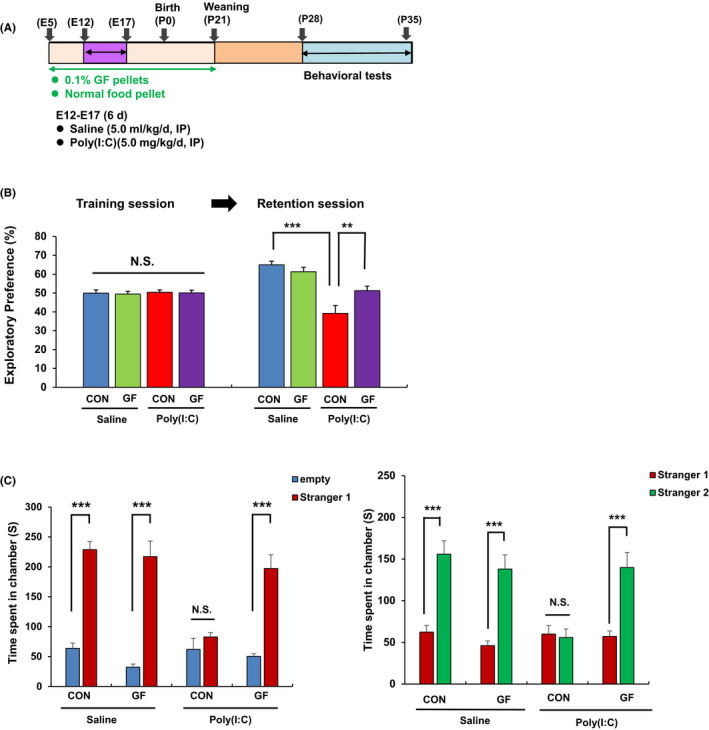
Effects of 0.1% GF food pellet on cognitive deficits and social interaction deficits in the juvenile offspring after prenatal poly(I:C) exposure. A, Schedule of treatment and behavioral tests. Saline (5.0 mL/kg/d) or poly(I:C) (5.0 mg/kg/day from E12 to E17) was injected into pregnant mice. Normal food pellets or 0.1% GF food pellets were given to pregnant mice from E5 to P21. Subsequently, normal food pellets were given to all mice from P21. Behavioral tests such as novel object recognition test (NORT) and 3‐chamber social interaction test were performed from P28 to P35. (B): NORT: There was no difference (two‐way ANOVA: poly(I:C): F_1,39_ = 0.122, *P* = .729, GF: F_1,39_ = 0.073, *P* = .789, interaction: F_1,39_ = 0.003, *P* = .954) between the four groups in the training session. In the retention session, two‐way ANOVA showed the results (poly(I:C): F_1,39_ = 37.73, *P* < .001, GF: F_1,39_ = 2.039, *P* = .161, interaction: F_1,39_ = 7.310, *P* = .010) between the four groups. In the retention test, the exploratory preference of poly(I:C) + GF food group was significantly higher than poly(I:C) + normal food group. **P < .05, ***P < .001 compared with poly(I:C) + normal food group. The value is expressed as the mean ± SEM (n = 10 or 11). (C): Three‐chamber social interaction test. Left: Two‐way ANOVA (empty: poly(I:C): F_1,31_ = 0.641, *P* = .429, GF: F_1,31_ = 4.423, *P* = .044, interaction: F_1,31_ = 0.939, *P* = .340. stranger 1: poly(I:C): F_1,31_ = 18.027, *P* < .001, GF: F_1,31_ = 6.887, *P* = .013, interaction: F_1,31_ = 10.371, *P* = .003). Right: Three‐way ANOVA (stranger 1: poly(I:C): F_1,31_ = 0.328, *P* = .571, GF: F_1,31_ = 1.576, *P* = .219, interaction: F_1,31_ = 0.761, *P* = .390. stranger 2: poly(I:C): F_1,31_ = 9.509, *P* = .004, GF: *F*_1,31_ = 0.434, *P* = .460, interaction: *F*_1,31_ = 10.202, *P* = .003). Data are shown as mean ± SEM (n = 8 or 9). ***P < .01. NS, not significant

We performed two behavioral tests (NORT, and 3‐chamber social interaction test) in juvenile offspring after MIA. Behavioral tests of juvenile offspring were performed at P28‐P35 after prenatal poly(I:C) injections (Figure 1A). In NORT, there was no difference between the four groups during the training session (Figure 1B). However, during the retention session, there was significant change among the four groups (Figure 1B). The exploratory preference of the poly(I:C) + normal food pellet group was significantly lower than that of the saline + normal food pellet group. Furthermore, the exploratory preference of the GF food pellet + poly(I:C) group was significantly higher than that of the normal food pellet + poly(I:C) group (Figure 1B).

In the three‐chamber test, juvenile offspring after MIA showed social interaction deficits compared to the control group (Figure 1C). Dietary intake of 0.1% GF food pellet significantly improved social interaction deficits in juvenile offspring after MIA (Figure 1C). The data suggest that MIA causes ASD‐like cognitive and social interaction deficits in juvenile offspring, and that dietary intake of 0.1% GF during pregnancy and lactation could prevent the onset of ASD‐like behavioral abnormalities in juvenile offspring after MIA.

### 3.2 Effects of dietary intake of 0.1% GF food pellets during pregnancy and lactation on cognitive deficits and reduction of PV immunoreactivity in the mPFC of adult offspring after MIA

**Figure 2:**
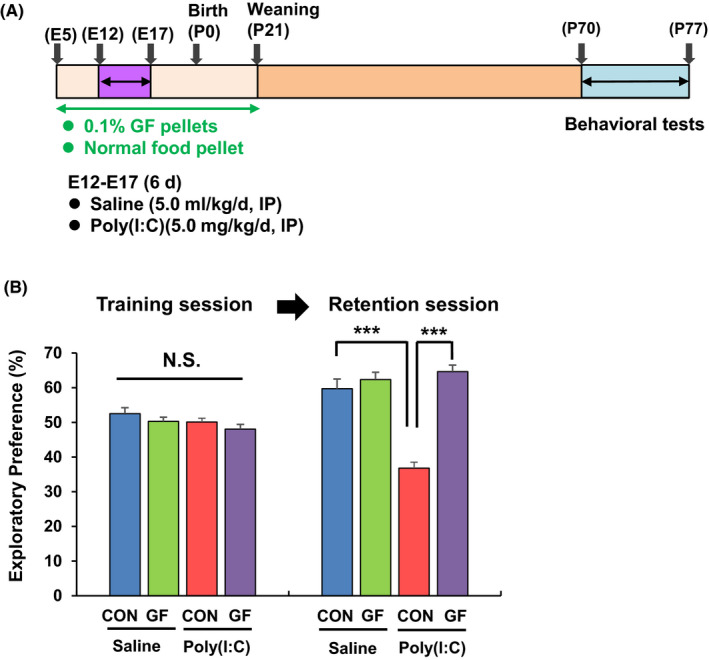
Effects of dietary intake of 0.1% GF on cognitive deficits in the adult offspring after prenatal poly(I:C) exposure. A, Schedule of treatment and behavioral tests. Saline (5 mL/kg/d) or poly(I:C) (5.0 mg/kg/d from E12 to E17) was injected into pregnant mice. Normal food pellets or 0.1% GF food pellets were given to pregnant mice from E5 to P21. Subsequently, normal food pellets were given to all mice from P21. B, NORT: There was no difference (poly[I:C]: *F*_1,38_ = 3.091, *P* = .087; GF: *F*_1,38_ = 2.563, *P* = .118; interaction: *F*_1,38_ = 0.003, *P* = .954) among the four groups in the training session. In the retention session, two‐way ANOVA showed the results (poly[I:C]: *F*_1,38_ = 23.88, *P* < .001, GF: *F*_1,38_ = 52.19, *P* < .001, interaction: *F*_1,38_ = 35.57, *P* < .001) between the four groups. In the retention test, the exploratory preference of poly(I:C) + GF food group was significantly higher than poly(I:C) + normal food group. ***P < .001 compared with poly(I:C) + normal food group. The value is expressed as the mean ± SEM (n = 9 or 11)

We investigated the effects of dietary intake of 0.1% GF food pellets during pregnancy and lactation on cognitive deficits and reduction of PV immunoreactivity in the mPFC of adult offspring after MIA (Figure 2A). In the training session of NORT, there was no difference among the four groups. However, in the retention session of NORT, the exploratory preference of the poly(I:C) + GF food pellet group was significantly higher than that of the poly(I:C) + normal food pellet group (Figure 2B).

**Figure 3:**
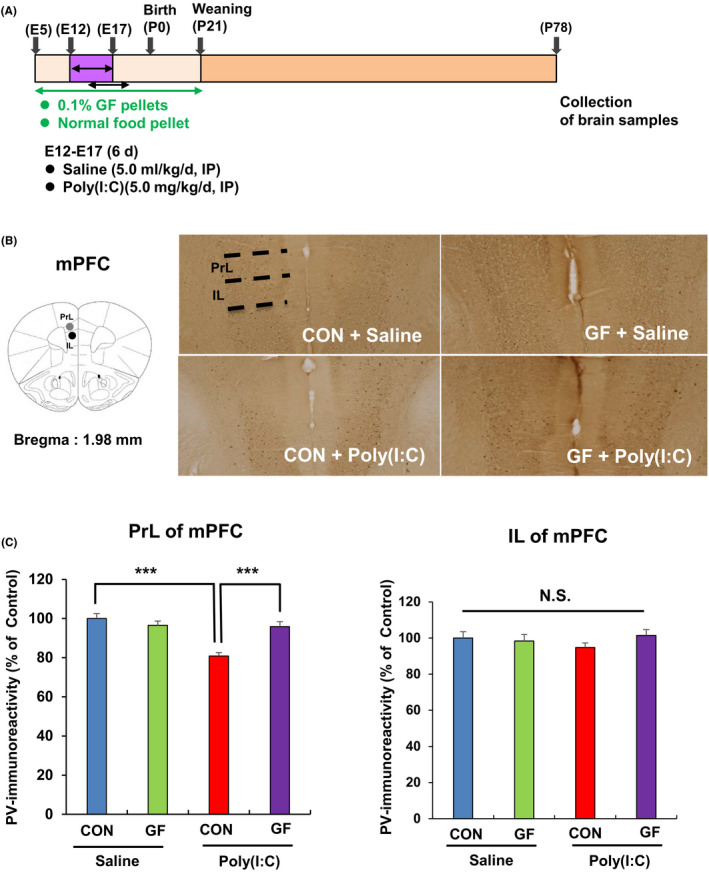
Effects of dietary intake of 0.1% GF on the reduction of PV immunoreactivity in the mPFC of adult offspring after prenatal poly(I:C) exposure. A, Schedule of treatment and behavioral tests. Saline (5 mL/kg/d) or poly(I:C) (5.0 mg/kg/d from E12 to E17) was injected into pregnant mice. Normal food pellets or 0.1% GF food pellets were given to pregnant mice from E5 to P21. Subsequently, normal food pellets were given to all mice from P21. On P78, brain was collected for PV immunohistochemistry. B, Brain atlas of PrL and IL regions of mPFC. The representative data of PV immunohistochemistry in the mouse brain. C, PrL of mPFC: Two‐way ANOVA showed the statistical results (poly[I:C]: *F*_1,22_ = 19.34, *P* < .001; GF: *F*_1,22_ = 6.49, *P* = .018; interaction: *F*_1,22_ = 16.67, *P* < .001). PV immunoreactivity in the PrL of mPFC of poly(I:C) + GF pellet group was significantly higher than that of poly(I:C) + control pellet group. IL of mPFC: Two‐way ANOVA showed the statistical results (poly[I:C]: *F*_1,22_ = 0.168, *P* = .685; GF: *F*_1,22_ = 0.409, *P* = .518; interaction: *F*_1,22_ = 1.475, *P* = .236) among the four groups. PV immunoreactivity in the IL of mPFC was not different among the four groups. The value is expressed as the mean ± SEM (n = 7 or 8). ***P < .001, compared with poly(I:C) + control pellet group. The value is expressed as the mean ± SEM (n = 7 or 8)

Furthermore, we performed PV immunohistochemistry at adulthood (11 weeks) (Figure 3A). PV immunoreactivity in the PrL (not IL) of the mPFC of the poly(I:C) + normal food pellet group was significantly lower than that in the saline + normal food pellet group. Furthermore, PV immunoreactivity in the PrL (not IL) of the mPFC of the poly(I:C) + GF food pellet group was significantly higher than that in the poly(I:C) + normal food pellet group (Figure 3B,C).

These findings suggest that supplementation of 0.1% GF food pellets during pregnancy and lactation prevented the cognitive deficits and the reduction of PV immunoreactivity in the PrL of the mPFC in adult offspring after MIA.

## 4 DISCUSSION

Here, we found that dietary intake of 0.1% GF food pellets during pregnancy and lactation prevented ASD‐ and schizophrenia‐like behavioral abnormalities and reduction of PV immunoreactivity in the PrL of the mPFC in offspring after MIA. Therefore, it is likely that supplementation with GF‐rich food in pregnant women with MIA (ie, higher inflammation) could have prophylactic effects on the development of neurodevelopmental disorders in offspring.

We found cognitive deficits of juvenile offspring after MIA, consistent with previous reports.^9^, ^10^, ^11^, ^12^ Given the role of cognitive impairment in ASD patients and subjects with a high risk for psychosis,^25^ it is likely that cognitive deficits may be a core behavioral deficit in juvenile offspring after MIA. Interestingly, dietary intake of 0.1% GF food pellet during pregnancy and lactation could block cognitive and social interaction deficits in juvenile offspring after MIA.

In this study, we also found reduction of PV immunoreactivity in the PrL, but not IL, of mPFC at adult offspring after MIA, consistent with the previous findings.^9^, ^10^, ^11^, ^12^ Interestingly, dietary intake of 0.1% GF food pellet during pregnancy and lactation could prevent reduction of PV immunoreactivity in the PrL of mPFC of adult offspring after MIA. Prenatal infection may contribute to the onset of neurodevelopmental disorders in their offspring.^1^, ^2^ Previously, we reported that dietary intake of 0.1% GF food pellet during juvenile and adolescence blocked the onset of cognitive deficits and reduction of PV immunoreactivity in the mPFC after repeated PCP administration.^19^ In addition, dietary intake of 0.1% GF food pellet during juvenile and adolescence blocked the onset of cognitive deficits and reduction of PV immunoreactivity in the mPFC of adult offspring after MIA.^12^ Furthermore, we also demonstrated that dietary intake of 0.1% GF food pellet might have prophylactic effects in chronic social defeat stress^22^ or inflammation,^23^ indicating a potent anti‐inflammatory action of 0.1% GF food pellet. Collectively, it is likely that dietary intake of 0.1% GF pellet has beneficial effects in several animal models of psychiatric disorders.

Sedlak et al^26^ reported that SFN increased the endogenous antioxidant glutathione levels in the blood and brain of healthy human subjects, indicating potent antioxidant effect of SFN. Interestingly, a placebo‐controlled, double‐blind, randomized study showed that supplementation of SFN had beneficial effects in young people with ASD.^27^ A subsequent follow‐up study showed that many parents and caregivers anticulated the beneficial effects of SFM, both during the intervention phase and in the ensuing 3 years.^28^ Taken all together, it is likely that supplementation of GF (or SFN)‐rich vegetables during pregnancy and lactation might have prophylactic effects on the development of neurodevelopmental disorders, such as ASD and schizophrenia.^7^

This manuscript has limitation. In this study, we did not investigate the tissue levels of GF and its metabolite SFN in the fetal brain. Therefore, it is unknown whether GF or SFN can affect directly altered cortical development of fetal brain after MIA. Further detailed study is needed.

In conclusion, the present data suggest that dietary intake of 0.1% GF during pregnancy and lactation could prevent the behavioral abnormalities in offspring after MIA. Finally, supplementation of GF (or SFN)‐rich vegetables in pregnant women with MIA or pregnant women at high risk for psychosis might reduce the risk of onset of neurodevelopmental disorders in offspring.

## CONFLICT OF INTEREST

Dr Hashimoto received speaker's honoraria from Murakami Farm (Tokyo, Japan) which sells sulforaphane‐rich vegetable. Drs. Ayumi Hirai, Shigenori Suzuki, and Hiroyuki Suganuma are employee of KAGOME which sells glucoraphanin‐related products as the supplement. The other authors declare no conflict of interest.

## AUTHOR CONTRIBUTIONS

KH is responsible for the design of the research and experiment and supervised the experimental analyses. KH wrote the paper. YF, AF, and TI performed behavioral experiments and immunohistochemistry. YF analyzed the data. AH, SS, and HS provided 0.1% GF food pellet. All authors read and approved this paper.

## ANIMAL STUDIES

All animal experiments were approved by the Animal Care and Use Committee of Chiba University.

## Supporting information

Fig S1‐S3

## Data Availability

The data that support the findings of this study are available in the Figure S1‐S3 of this article.
